# Obstacles to global implementation of CAR T cell therapy in myeloma and lymphoma

**DOI:** 10.3389/fonc.2024.1397613

**Published:** 2024-07-19

**Authors:** Fernando J. Medina-Olivares, Andrés Gómez-De León, Nilanjan Ghosh

**Affiliations:** ^1^ Facultad de Medicina y Hospital Universitario Dr. Jose Eleuterio Gonzalez, Universidad Autonoma de Nuevo Leon, Monterrey, Mexico; ^2^ Atrium Health Levine Cancer Institute, Wake Forest School of Medicine, Charlotte, NC, United States

**Keywords:** barriers, CAR T, lymphoma, myeloma, access

## Abstract

Chimeric Antigen Receptor T-cell (CAR-T) therapies are transforming the treatment of B-cell lymphoproliferative disorders and multiple myeloma, yet global access challenges and barriers for their implementation persist. Global access disparities persist, particularly for persons living in low and middle-income countries and for underserved populations in high income countries. In this review we address patient-related factors including age, comorbidities, fitness, race and ethnicity, and geographic location for CAR-T access. Also, we review disease-related and health system barriers like disease biology, potential for short and long-term toxicity, insurance access, referrals, supply and manufacturing, regulation, costs and treatment center capacity. Lastly, alternatives for overcoming these barriers exemplified by research efforts worldwide are discussed, emphasizing the need for a multifaceted approach from all stakeholders to improve global accessibility and ensure equitable access and improved outcomes for patients worldwide.

## 1 Introduction

Chimeric antigen receptor T-cell therapies (CAR-T) have revolutionized the management of persons with relapsed/refractory B-cell lymphoproliferative disorders (1) The first generation of CAR-T cells were not clinically effective due to low persistence (2) Subsequently, the emergence of second-generation CAR-T cells designed to target the antigen CD19 with CD28 or 4-1BB co-stimulatory domains coupled with CD3 ζ improved CAR T cell *in vivo* persistence and efficacy, leading to their establishment as a novel treatment modality for patients with B-cell non-Hodgkin Lymphomas (3) In 2013, reports of CAR T-cell therapies directed against the B-cell maturation antigen (BCMA) aimed for persons with multiple myeloma (MM) emerged. Since 2017, six CAR T products have been approved for various indications in relapsed or refractory B cell non-Hodgkin lymphoma or multiple myeloma (4).

In 2023 the Center for International Blood and Marrow Transplant Research (CIBMTR) reported data from 214 centers documenting 10,976 patients who have undergone various form of cellular therapies. Among this cohort, 6,646 patients underwent treatment for lymphoma, and 1,401 received treatment for ALL. Since their inception, the cell therapy field has undergone significant advances, but still many challenges in terms of delivery and applicability remain. Access to CAR-T cell therapy remains limited to some populations and in this review, we aim to document obstacles for their implementation and potential solutions to overcome them.

## 2 Patient-related factors

The scientific rationale for imposing specific chronological age restrictions in clinical trials has not been firmly established. However, it is noteworthy that 64% of trials have implemented upper age limits (5). Outside of clinical trial settings, the use of CAR-T cell therapy has been shown to be effective in older adults with NHL (6). Also, patients with myeloma may not be fit for autologous transplantation but may be considered eligible for CAR-T (7).Thus, rather than using chronological age cutoffs, a holistic assessment guided by a comprehensive geriatric assessment in older adults has been recommended to identify patients who may be at risk of complications and identify areas of opportunity for early intervention (8). On the other end of the spectrum, except for tisagenlecleucel, all approved CAR-T cells for treating persons with lymphoma and myeloma are approved for adults exclusively.

Globally speaking, country of residence perhaps remains the most important patient-centered predictor of access to CAR-T cell therapy. This treatment remains limited to only a few countries worldwide, mostly high-income countries in North America, Europe, Asia, and the Pacific, and in selected middle-income countries like China, Brazil, and India. Access runs in parallel to that of hematopoietic cell transplantation, as most procedures are performed in these world regions as well, given that it requires a similar infrastructure to CAR-T. Substantial expertise, resources, and a comprehensive network of specialists across various medical domains are necessary requisites. Most patients remain limited to what their health system has to offer with international travel only limited for the wealthy.

In China significant investment in biotechnological research and infrastructure has occurred, facilitating the rapid advancement and scaling of CAR T cell technologies. On December 11, 2017, the Chinese regulatory agency approved the first Investigational New Drug application for CAR T therapy from Nanjing Legend Biotechnology Co., Ltd. In recent years, additional technology start-ups, like Cellular Biomedicine Group, and Fosun Kite Biotechnology Co., Ltd., have emerged seeking to manufacture CAR T cells for cancer treatment. CARsgen Therapeutics Holdings Limited, specializing in innovative CAR T-cell therapies for hematologic and solid tumors, announced that the National Medical Products Administration (NMPA) of China has approved their New Drug Application (NDA) for zevorcabtagene autoleucel. This autologous CAR-T product targets BCMA and is approved for treating adult patients with relapsed or refractory multiple myeloma who have previously undergone at least three lines of therapy, including a proteasome inhibitor and an immunomodulatory agent.

A survey among groups of hematologists and transplant recipients in Latin America (LA) to assess potential CAR-T initiatives revealed active commercial studies in Brazil and Argentina. In Brazil and Mexico, projects aimed at the development of CAR-T therapy through partnerships with international academic institutions are ongoing (9,10). India’s inaugural approved CAR-T, actalycabtagene autoleucel, represents a pioneering product developed in a low middle income country (LMIC) aimed at segments of society at an affordable cost (11). In countries where CAR T-cell therapies are commercially available, every facet poses obstacles to accessibility for vulnerable populations. The treatment necessitates administration at a specialized center, requiring patients to stay in proximity for at least one month. The timeframe for referral is limited, potentially exacerbating existing disparities in access and biases. Moreover, the therapy is costly, involving resource-intensive logistics, and insurance-related hurdles (12). A retrospective study in the United States conducted an evaluation of the accessibility of CAR T-cell therapy, considering both provider and patient locations and compared the travel distances of individuals undergoing CAR T-cell therapy (cohort A) from their residences to one of the 64 Foundation for the Accreditation of Cellular Therapy-accredited centers, with those of patients receiving alternative disease-based treatments (cohort B). The results indicated that patients residing in the Southern region of the United States covered significantly greater distances in travel compared to their counterparts in other regions, ranging from 17.2 to 46.7 miles versus 0.3 to 14.5 miles (13). Additionally, limited resources at referral centers further compound logistical challenges not addressed by patient assistance programs. A study conducted by Faruqi et al., which retrospectively assessed the impact of demographics and obesity on CAR T-cell therapy outcomes, found that factors such as race, ethnicity, and BMI did not have a significant influence on the effectiveness of CAR T-cell therapy or the occurrence of neurotoxicity. However, in the clinical trial environment, additional systemic barriers for racial and ethnic minority groups contribute to their underrepresentation, as clinical trial participants are mostly non-Hispanic white people, with less clinical trial openings in regions of the United States with a higher percentage of Black residents (14). Similarly, the Pediatric World CAR Consortium has reported lack of access and fewer treatments in Black/African American patients (5.5% of the 200-patient cohort) with a greater number of prior treatment lines, more relapses, and higher rates of prior hematopoietic cell transplantation before receiving CAR T cell therapy (15).

Another patient-related barrier is immune fitness. A retrospective, multicenter, international study led by Lacoboni et al. assessed 370 patients with relapsed/refractory large B-cell lymphoma treated at 7 sites using commercially available CAR-T cell products showed that those receiving bendamustine prior to apheresis (74%) had a lower and delayed absolute peak expansion of CAR-T cells after infusion compared to the bendamustine-naïve control group. Bendamustine-containing regimens prior to CAR T-cell therapy may negatively impact T-cell numbers and composition at apheresis and subsequent CAR T-cell expansion. Patients with recent (<9 months) exposure to bendamustine and large B-cell lymphoma show worse outcomes after CAR T-cell therapy compared to those with earlier exposure to this chemotherapeutic agent (16).

To overcome immune exhaustion, the use of healthy donors for cell manufacturing is logical. Off-the-shelf readily available cell therapies that can be used without the need for patient-specific customization or harvesting of the patient’s own cells is a vision for the future. In allogeneic CAR T cell recipients the risk of graft-versus-host disease is low, and the utilization of adoptive CAR T therapy using donor-derived cells has proven to be effective (17). The ability to genetically modify T cells to eliminate the endogenous T cell receptor, thereby enabling the use of T cells derived from healthy donors without the risk of graft-versus-host disease, has been studied in several platforms, including using T cell gene editing and umbilical cord-derived NK cells (18–20). Unlike autologous therapies that require individualized cell harvesting and manufacturing processes for each patient, off-the-shelf cell therapies can be mass-produced, potentially reducing costs and wait times. The allogeneic approach can lead to a faster production timeline, as the cells can be pre-manufactured and stored for use when needed.

## 3 Disease biology

Fast and uncontrolled growth of cancer cells can pose a significant challenge in the context of CAR T. Patients may undergo lymphocyte apheresis but experience an event of disease progression before the cells are manufactured and infused. A systematic analysis highlights a lack of consistent reporting regarding dropouts in published CD19 and BCMA CAR-T trials, specifically those linked to disease progression or manufacturing failure, which occur after enrollment but before the initiation of therapy (21). Additionally, the reasons why patients who do not receive CAR-T after enrolling are often insufficiently documented. In instances where such information is provided, a noticeable reduction in the number of patients is commonly observed from the initial enrollment stage to the actual administration of CAR-T therapy that affects the effectiveness of the treatment. For bridging therapy (BT), conventional treatments like chemotherapy or radiation therapy to stabilize the disease, is considered safe, and can achieve responses before cells are infused. A study focused on 375 adult patients with diffuse large B-cell lymphoma (LBCL) examined the modality and response of BT in relation to outcomes following the administration of axicabtagene ciloleucel (Axi-cel) or tisagenlecleucel (Tisa-cel). Most patients underwent BT using chemotherapy (57%) or radiotherapy (17%). The findings indicated that BT was well-tolerated by patients, with minimal morbidity or mortality observed with a 42% reduction in the risk of progression or death after CD19 CAR-T therapy (22).. Patients undergoing autologous chimeric antigen receptor T cell (CAR-T) therapy for multiple myeloma (MM) might necessitate BT prior to CAR-T infusion to acheive a certain level of disease control. Alkylators, like cyclophosphamide (Cy), are commonly incorporated into treatment protocols. The intensity of BT may depend on the disease kinetics and various regimens have been used (23).

Central nervous system (CNS) and other extramedullary sites of disease involvement can potentially pose challenges or limitations in the effectiveness of CAR T-cell therapy. These sites were thought to be difficult to reach limited by the blood-brain barrier or unique microenvironments, impacting cells’ ability to access and eradicate cancer cells, thus the rationale for the exclusion of persons with CNS involvement in the JULIET and ZUMA-1 trials. Recent studies including a systematic review and experiences outside clinical trials have shown CAR-T to be effective in central nervous system lymphoma (24, 25). Similarly, case reports reporting positive experiences in myeloma have also been published (26).

Certainly, the overall prognosis for extramedullary multiple myeloma remains bleak, and conventional treatments have shown limited efficacy. A study from China highlighted that extramedullary disease (EMD) significantly impacted the prognosis of patients undergoing anti-BCMA CAR-T therapy for relapsed/refractory multiple myeloma (RRMM). Interestingly, patients with extra-medullary myeloma (EMM) exhibited lower rates of cytokine release syndrome (CRS) compared to those without EMM (27). Systematic analyses indicate promising initial response rates with CAR-T therapy; however, these responses tend to be transient (28).. Pan et al. conducted an analysis focusing on RRMM patients treated with CAR-T cell therapy in a clinical trial. Notably, half of the patients experiencing relapse post-CAR-T therapy had EMD. Strikingly, despite radiographically negative EMD following CAR-T treatment, most patients with initial EMD experienced subsequent relapse characterized by extramedullary disease (29).

Reduced target antigen expression is another way disease can evade CAR-T. CD19-targeted chimeric antigen receptor T cells (CD19-CAR) and blinatumomab have shown efficacy in inducing remission among relapsed or refractory B-cell acute lymphoblastic leukemia (ALL) patients. However, there’s a notable association between these therapies and CD19 antigen modulation. Limited data exist concerning how prior exposure to blinatumomab might affect subsequent CD19-CAR outcomes. Blinatumomab use has been linked to a reduction in CD19 expression. In cases where CD19 expression decreased or altered post-blinatumomab treatment, there was a heightened risk of relapse post-CD19-CAR therapy with CD19-negative disease. Several factors, such as inherent T-cell dysfunction, resistance to immunotherapy, or the adverse impact of extensive prior treatments, could contribute to the poorer outcomes observed in patients previously exposed to blinatumomab. This effect seems particularly pronounced within a patient population heavily treated with various therapies (30).

Resistance or relapse due to the absence of the target antigen (CD19 loss or downregulation) has been extensively researched. Multiple studies have indicated that instances of CD19-negative relapses occur in approximately 9% to 25% of cases of B-cell acute lymphoblastic leukemia treated with CAR T cell therapy (31). Relapses in some cases exhibit either a lack of antigen presence or lower antigen levels. In the KarMMa study, approximately 6% of patients who relapsed showed antigen loss upon immunohistochemistry assessment, while around 4% experienced serum antigen reduction measured by soluble BCMA (32). Although BCMA antigen loss occurs infrequently (4-33%) in patients treated with anti-BCMA CAR-T, one approach is to target BCMA using CARs with higher affinity or tighter binding, like biparatopic binding domains. However, this strategy may lead to more on-target toxicity, as seen in recent reports of Parkinsonian symptoms in at least six patients treated with anti-BCMA CAR T cells (33).. Various methods exist for engineering multi-specific T-cell products to counter antigen escape, such as utilizing single bicistronic vectors expressing two CARs, employing tandem vectors housing a single CAR with dual binder sequences, or co-transducing CAR T cells with separate CAR-encoding vectors (31) Multiple targets are under investigation in multiple myeloma, extending beyond BCMA to include CD19, CD38, GPRC5D, CD1, and SLAMF7 (34). Co-targeting studies of CD19/BCMA showcased a robust overall response rate of 95%, accompanied by complete response rates ranging from 16% to 57% (35) Similarly in lymphoma combinations of CD19 with CD22 are under study (36).

To overcome aggressive disease biology, several modifications to fine-tune CAR T cell design are underway. The ability to deactivate or eliminate T cells as needed, redirect universal CAR T cells using a soluble antigen recognition domain represent exciting and significant developments (37). Studies combining CAR T therapy with other treatments, such as checkpoint inhibitors, other monoclonal antibodies and small molecules are ongoing to enhance their effectiveness and durability (38).

## 4 Adverse events

Cytokine release syndrome (CRS) is a common and potentially severe side effect of CAR T therapy, characterized by the release of inflammatory cytokines, leading to fever, hypotension, and organ dysfunction. This phenomenon arises due to a hyperactive systemic immune reaction orchestrated by T cells, B cells, NK cells, and monocytes, resulting in the release of a substantial quantity of inflammatory mediators, including cytokines and chemokines (39). Managing CRS is crucial but challenging. Identifying pre-infusion risk factors linked to the occurrence and severity of subsequent CRS is crucial for pinpointing high-risk patients who could benefit from early intervention studies. Prophylaxis for CRS involves strategies aimed at preventing or mitigating the severity of CRS in individuals at risk, particularly those undergoing certain immunotherapies or treatments known to trigger CRS. Before starting therapies like CAR T-cell therapy, patients undergo risk assessment to identify factors that might predispose them to CRS. Using corticosteroids before or during treatment can proactively regulate the immune response, potentially lessening the severity of CRS. Tocilizumab, an IL-6 receptor antagonist, has been used to prevent or manage CRS by obstructing the IL-6 pathway, a key player in the cytokine release cascade. Rigorous patient monitoring during and post-therapy enables timely intervention upon detecting initial signs or symptoms of CRS. This comprehensive monitoring entails observing vital signs, conducting laboratory tests, and being attentive to patient-reported symptoms.

Neurological manifestations have been documented under the acronym ICANS (Immune Effector Cell-Associated Neurotoxicity Syndrome) and encompass a range of symptoms, including but not limited to headaches, cognitive disorientation, restlessness, seizures, tremors, language difficulties, comprehension challenges, aphasia, cranial nerve irregularities, and visual hallucinations (40).. Understanding and managing this toxicity remain areas of active research. Suggested factors that might predispose individuals to developing ICANS include pre-existing neurological conditions, previous occurrence of CRS, increased doses and peak expansion levels of CAR-T cells, elevated tumor burden during CAR-T infusion, reduced platelet counts at infusion, and elevated levels of inflammatory markers such as C-reactive protein (CRP) or ferritin, as well as certain cytokines like interleukin IL-1, IL-6, IL-10, and interferon-gamma. The current established approaches to treatment primarily involve corticosteroids and supportive care, with the specific role of anti-cytokine therapy yet to be precisely determined. Within the ZUMA-1 study, various safety cohorts explored alternative management or preventative strategies for CRS/ICANS and were compared against those in the pivotal cohorts. Earlier administration of corticosteroids or their prophylactic use appeared to reduce both the occurrence and severity of ICANS without visibly affecting treatment efficacy. A promising avenue of exploration involves inhibiting IL-1 signaling through the IL-1 receptor antagonist anakinra, based on preclinical research and emerging reports demonstrating its utilization in treating ICANS that doesn’t respond to steroids. Access to CAR T is limited to centers which have the expertise in managing these adverse events. Establishing an effective prophylactic strategy has enabled safer administration, facilitating the delivery of these therapies with curative intent at higher doses to a broader patient population. This broader administration could encompass individuals for whom current risks are considered higher due to age or existing medical conditions, thus allowing the benefits to potentially outweigh the risks. Other relevant concerns are the long-term risks of genotoxicity.

## 5 Costs

Drug costs, whether within the United States or on a global scale, have witnessed significant escalation in the last two decades. Presently, the median initial price for cancer medications in the United States surpasses $155,000 USD annually (41).. The high price tag makes many drugs inaccessible to patients and poses a difficult challenge to healthcare systems worldwide. CAR T therapy is no exception and is extremely expensive. Gilead set the initial price of Yescarta (axicabtagene isoleucel) in the United States at $373,000, but it has since increased to $424,000. Remarkably, this cost is twice that of the therapy in Japan. Novartis established the price of tisagenlecleucel at $475,000, solely for the drug products, excluding expenses related to leukapheresis, lymphodepletion therapy, and the potential adverse effects associated with CAR-T immunotherapy. Llisocabtagene maraleucel, another CAR-T therapy approved for lymphoma, is priced at $410,300. Idecabtagene vicleucel, developed by Celgene (now part of Bristol Myers Squibb), is priced at $419,500. Brexucabtagene autoleucel, priced at $533,523 per patient. Lastly, Ciltacabtagene autoleucel, introduced by Janssen, is listed at $465,000. Reimbursement strategies involve navigating complex financial considerations associated with these innovative and expensive treatments. First, patients receiving CAR-T therapy often must rely on health insurance to cover a significant portion of the treatment costs. Insurance plans may vary in terms of coverage, and patients and healthcare providers need to navigate the specifics of each plan to determine the level of reimbursement. Healthcare providers, pharmaceutical companies, and payors engage in negotiations to determine the reimbursement rates for CAR-T therapies. These negotiations may involve discussions on pricing, patient access, and the overall value of the treatment. Some reimbursement strategies for CAR-T therapies include outcomes-based agreements. In these arrangements, payment may be contingent on the treatment’s effectiveness, measured by predefined clinical outcomes (12, 42). This approach aligns reimbursement with treatment success and patient outcomes. Pharmaceutical companies often establish patient assistance programs to help individuals access CAR-T therapies in high income countries. These programs may offer partial financial assistance, copay support, or other forms of aid to alleviate the financial burden. In high income countries, government healthcare programs play a role in reimbursing for CAR-T therapies. A recent study from the University of Nebraska recently demonstrated that patients with private insurance often need single case agreements (SCA) and endure significant longer delays in time from intent to CAR-T to receiving the CAR-T product compared to patients with government insurance (43). Generating and using real-world evidence on the long-term effectiveness and economic impact of CAR-T therapies can support reimbursement discussions. This evidence may include data on real-world patient outcomes and the overall value of the treatment. Navigating reimbursement strategies for CAR-T therapies requires collaboration among stakeholders, including healthcare providers, pharmaceutical companies, payers, and regulatory bodies. Commercial CAR-T therapies have predominantly found distribution in key regions such as North America, Western Europe, and the Western Pacific. Conversely, in Latin America, particularly in countries like Brazil, the use of CAR-T therapies has been isolated, primarily due to the regulatory background and capacity of the health system to bear the logistical, clinical, and financial burden of such treatments.

## 6 Manufacturing chain and supply

The allocation of manufacturing slots for CAR-T therapy includes the strategic scheduling and allocation of production resources to meet the demand for these complex and personalized treatments. Manufacturers need to assess and plan their production capacity based on the anticipated demand for CAR-T therapies. This involves considering factors such as the number of patients expected to undergo treatment and the production capabilities of manufacturing facilities. The allocation of manufacturing slots is influenced by the enrollment of patients in clinical trials and later by the prescription patterns of CAR-T therapies. Manufacturers should work closely with institutions to schedule patient treatments, aligning them with available manufacturing slots and reducing the “brain-to-vein” time. The supply chain for CAR-T therapies involves various components, including the collection of patient cells, transportation, and manufacturing. Adherence to regulatory requirements is paramount in the manufacturing of CAR-T therapies. Manufacturers must ensure that their processes comply with regulatory standards, and manufacturing slots are allocated with consideration for regulatory timelines and approvals. In addition, rigorous quality control measures are essential in CAR-T manufacturing. Allocation of manufacturing slots considers the time required for thorough quality checks and assurance procedures to guarantee the safety and efficacy of the final product. Efficient use of manufacturing resources, including personnel, equipment, and facilities, is a critical factor in slot allocation. Close collaboration between manufacturers and the treatment team is crucial in the slot allocation process and may represent a significant barrier to access for individuals with lymphoma and multiple myeloma (44). Following the popularity of the therapy and limited treatment alternatives in multiply treated myeloma patients bottlenecks in the manufacturing space have been reported (45). The ‘vein to vein’ duration, referring to the time between apheresis and infusion of CAR T cells, stands another critical factor influencing patient outcomes (46). Prolonged vein-to-vein timelines create issues due to the rapid disease progression, impacting the eligibility of end-stage patients for CAR T treatment. Companies actively explore strategies to minimize vein-to-vein time, such as optimizing time-constrained quality control processes, aimed at gaining a competitive edge (47). An alternative strategy to reduce vein-to-vein time, lower production costs, and enhance scalability involves exploring decentralized CAR-T production models. This approach entails producing CAR-T products within academic hospitals using GMP-grade facilities or employing automated CAR-T manufacturing systems like the Miltenyi Prodigy or Lonza Cocoon incubator. In Switzerland, institutions have ventured into manufacturing CAR T cell therapies at a cost ranging from US$150,000 to US$200,000, approximately half the price of most approved CAR-T cell therapies. However, in the United States, regardless of production site (academic or industry), CAR-T cell therapies are subjected to identical regulatory processes and pre-market approval as drugs, constraining their widespread implementation (12). Addressing this barrier involves exploring the development of allogeneic CAR-T cells. Allogeneic CAR T-cell therapy holds the potential to establish repositories of cryopreserved cells within individual hospitals, enabling quicker timelines for patients. Advances in genome editing tools, via CRISPR/Cas9, can allow us to overcome the two main limitations of allogeneic CAR T cells i.e., graft-vs.-host disease and host allorejection. Advancing next-generation allogeneic CAR T cells is a focal point of ongoing research to tackle these challenges (48). These manufacturing challenges have led to notably high fixed expenses, resulting in reluctance to extend CAR T therapy to a wider group of patients. Timely communication about patient schedules, treatment plans, and any unforeseen challenges can help streamline the manufacturing process and timely availability of the CAR T cell product.

The T2EVOLVE initiative, part of the Innovative Medicine Initiative (IMI) consortium, aims to accelerate the development of CAR and TCR engineered T cell therapies within the EU. By utilizing tools available in the current EU regulatory framework, T2EVOLVE strives to support an iterative and adaptive learning approach across various product versions that share similar design elements or are based on the same platform technology. As the understanding of the connections between product quality attributes, manufacturing processes, clinical efficacy, and safety improves through both development and post-licensure phases, new opportunities are arising to streamline regulatory submissions, enhance clinical studies, and extrapolate data across different product versions, thereby minimizing the need for repetitive studies.

## 7 Hospitals and pathways of care

The burden on referral centers, including limited capacity, a shortage of trained personnel, and a constrained number of hospital beds, poses challenges in the context of inpatient CAR-T therapy. In response to this, one potential solution is the implementation of outpatient CAR-T therapy. The scarcity of specialized centers, skilled personnel, and available hospital beds dedicated to CAR-T therapy can impede the timely and efficient administration of treatments. During the initial stages of development of CAR-T therapy, inpatient hospital stay due to monitoring and management of potential side effects was required in all cases. This strategy can strain hospital resources and limit the number of patients who can receive treatment. Implementing outpatient CAR-T therapy offers a potential solution to address these challenges minimizing the need for prolonged inpatient stays. This approach enhances treatment accessibility, reduces the burden on inpatient facilities, and can streamline the treatment process. Outpatient management of lymphoma and myeloma patients has become standard. Evidence from trials indicates that a notable portion of patients treated in outpatient settings eventually require hospitalization due to severe side effects, notably CRS and ICANS. Data on the safety of outpatient CAR T-cell therapy has been reported for specific products like tisa-cel and liso-cel. Initial reports demonstrate outpatient infusions using tisa-cel in approximately 24% to 27% of patients in studies for B-cell ALL and DLBCL, respectively (49). The University of Pennsylvania also reported relatively safe outpatient administration of tisa-cel, with a 31% admission rate post-infusion (50) Moreover, Bachier et al. indicated that patients receiving liso-cel could be safely monitored in outpatient settings, with 59% requiring hospitalization post-infusion within a median of 5.5 days, and only 8% hospitalized within three days of infusion (51). Hence, an evaluation of a patient’s risk for these side effects, the onset time of CRS and ICANS, and the availability of trained providers and caregivers is essential to determine outpatient suitability. To mitigate risks, outpatient facilities must establish comprehensive safety protocols and possess adequate resources to manage CRS and ICANS, encompassing both physical resources (like hospital access and medications) and proper training of clinical staff.

In Latin America, implementing outpatient CAR T-cell therapy would require adaptation to regional healthcare systems. Establishing specialized centers with robust safety measures and well-trained staff could facilitate safe outpatient administration. This approach demands careful consideration of resource allocation and training programs tailored to the region’s healthcare landscape to ensure effective and safe outpatient treatment. It is essential to carefully assess patient safety and the feasibility of outpatient management, considering factors such as the specific CAR-T construct used, the patient’s health status, and the potential for adverse reactions. Close monitoring and coordination with healthcare providers are crucial elements of successful outpatient CAR-T therapy implementation.

Products manufactured in the point-of-care from non-commercial academic sources like University Hospitals offer flexibility in patient treatment and can come with lower costs for health systems. Notably, in Spain, local vectors which encode a CAR against CD19 and BCMA product have been developed (ARI-001 and ARI-002, respectively) with cell manufactured implemented with the Miltenyi Prodigy^®^ manufacturing platform. The anti-CD19 product has obtained the approval of the Spanish regulatory agency and has been given a PRIME designation by the EMA (52). Spanish patients who are not candidates for commercial therapies may receive academic CAR-T in several institutions that have incorporated this alternative into their care pathway. This product and its point of care manufacturing platform is under study in several countries in Europe, Latin America, Asia, and is posed to be the first academic product to compete with products distributed by pharmaceutical companies. Recently, the results of the Spanish BCMA product have been published showing comparable safety and efficacy to commercially available products in RRMM (53). While not strictly an academic effort, the Cocoon system has been studied through the implementation of local CAR-T manufacturing closed systems in several centers in real time with monitoring by the sponsor in the Galapagos trials (54). Access to effective vectors becomes a limiting step for institutions aiming for POC manufacturing and academic CAR-T. Caring Cross, a nonprofit organization, is actively driving progress in CAR T accessibility by spearheading a humanitarian licensing approach. This strategy aims to streamline commercialization processes while concurrently fostering sustainable access in low- and middle-income countries (LMICs) (55) This initiative is designed to encourage widespread standardization across the industry, laying a robust foundation for academic institutions and early-stage startups to manufacture lentiviral and Adeno-associated virus (AAV) vectors that align with potential clinical development. Even with an accessible vector, POC CAR-T manufacturing is a challenging procedure. It requires specialized manufacturing facilities, skilled personnel, and infrastructure for transportation and administration. Assessment of the apheresis service and cell processing laboratory is necessary to ensure compliance with release product specifications. This evaluation may entail acquiring extra equipment for product collection and final product storage, improving the capacity to transport and receive cellular products, and ensuring an adequate workforce for executing these procedures (56). Developing and maintaining these resources can be a barrier to its widespread adoption. Furthermore, the low numbers of hematopoietic cell transplants performed in LMICs reflect the lack of necessary infrastructure and resources that will likely impact the capacity for implementation of academic CAR-T therapy.

Even in places with established transplant programs, manufacturing CAR T-cells is a complex and time-consuming process. This delay can be critical for patients with aggressive diseases who need rapid intervention. Strategic investments in optimizing the CAR T-cell manufacturing process are crucial for reducing production time. Leveraging automation and advanced techniques can significantly expedite this phase. Establishing centralized CAR T-cell manufacturing facilities enhances efficiency and widens the reach to serve a larger patient population promptly, minimizing wait times and expediting treatment. Rigorous adherence to quality control standards during CAR T-cell production is imperative for ensuring patient safety. Delays may arise if the manufactured CAR T-cells fail to meet these stringent standards, necessitating re-manufacturing. One of the key objectives in the manufacturing process is to invest in a robust quality control system to minimize the likelihood of non-conforming products. This involves comprehensive testing of cells for purity, potency, and safety. The implementation of real-time monitoring systems during manufacturing detects issues early, allowing for swift corrective actions. Ensuring well-trained manufacturing staff adheres to standardized operating procedures is paramount. Proper documentation of the manufacturing process aids in identifying and rectifying issues promptly. Having backup manufacturing capacity available is essential to quickly address non-conforming products or unforeseen delays, ensuring a consistent supply of CAR T-cell therapies.

In LMICS several additional challenges have hindered the widespread adoption of CAR-T. Lack of legislation and regulation require complex frameworks and represent formidable barriers to overcome. The absence of clear guidelines for the approval, manufacturing, importation, exportation, and clinical use of CAR-T products adds further complexity. Without them, the conduct of clinical trials cannot occur, and authorization of commercial products remain a dream. The example of India could be followed, where actalycabtagene autoleucel, a CD19 product received approval from the Central Drug Standards Control Organisation (CDSCO) of India. This approval positions ImmunoACT to spearhead the development of indigenous CAR-T cell therapy within the country. NexCAR19 will be priced at $36,000-$48,000 USD, representing an approximately one-tenth of the cost compared to commercially approved therapies worldwide. This competitive pricing could potentially attract patients from neighboring counties who don’t have access to CAR T, offering access to an exclusive therapy at a substantially lower cost. Nevertheless, it would remain financially inaccessible for the vast majority of individuals within India. Even after authorization, securing reimbursement for CAR-T treatments within healthcare systems becomes the next challenge, primarily due to limited financial support and reimbursement mechanisms for these advanced and expensive therapies in the context of limited resources, healthcare expenditures and alternative priorities of care.

Therefore, cost-effectiveness studies performed across different settings are needed.

Addressing these obstacles necessitates a comprehensive strategy involving collaboration among regulatory bodies, healthcare systems, and international organizations and institutions. The focus should be on developing transparent regulatory pathways, enhancing capacity for HSCT procedures, and establishing mechanisms for the approval and reimbursement of CAR-T therapies within resource-constrained financial environments. Additionally, initiatives for education and awareness are vital to ensure that healthcare professionals and policymakers in LMICs are well-informed about the potential benefits and challenges associated with CAR-T therapies.

## 8 Conclusion

**Figure 1:**
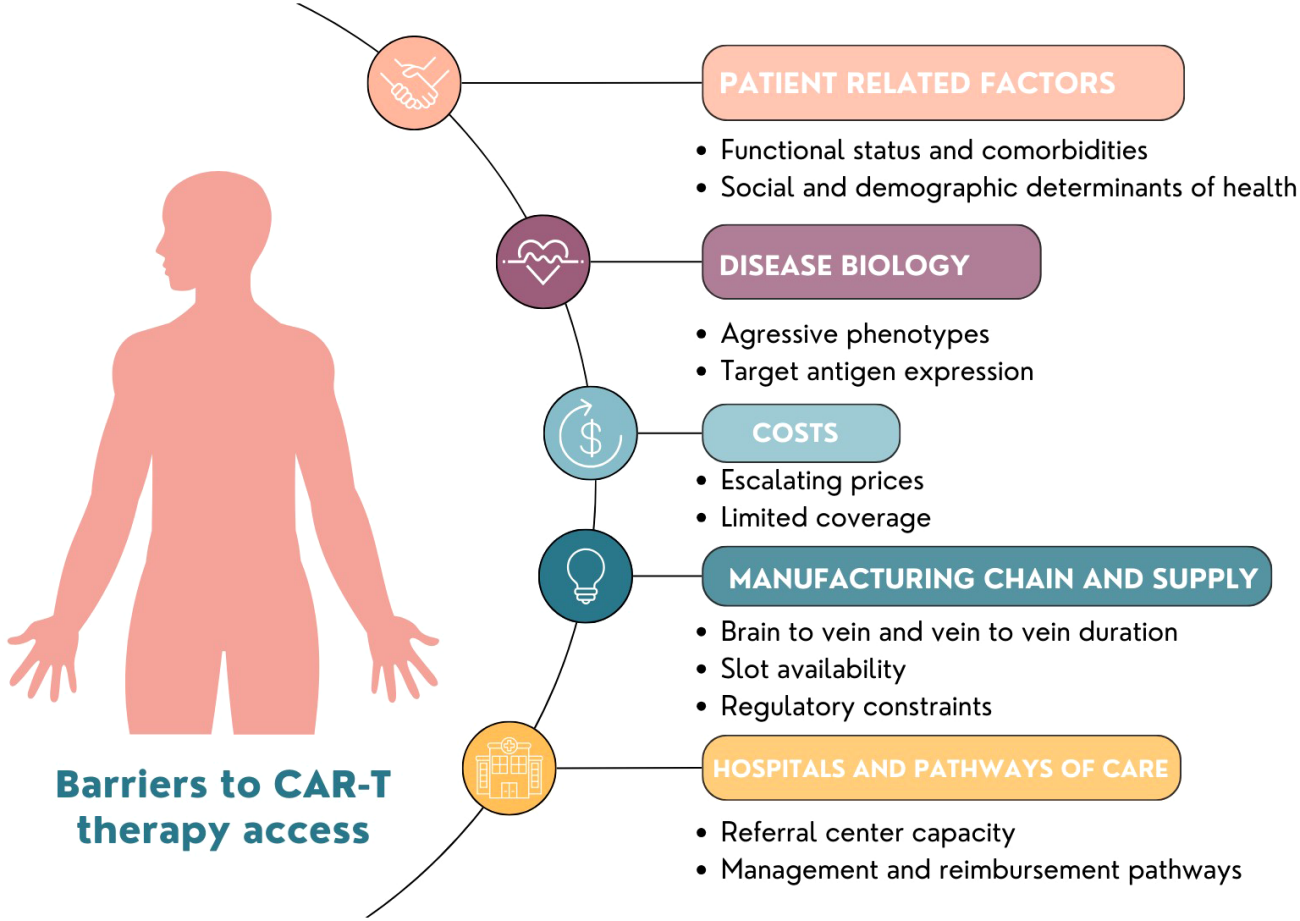
Barriers to Global Implementation of CAR-T.

While CAR T therapy has made significant strides in recent years, addressing these obstacles is essential to make this revolutionary treatment more accessible, safe, and effective for a broader range of cancer patients. Studies reporting current access to CAR T and using specific interventions to improve access are needed to overcome these challenges. In addition to reporting the outcomes of patients who receive CAR T, studies should report the number of patients who were eligible for CAR T but were not able to receive them and detail the barriers for receiving CAR-T. In summary, overcoming these obstacles in CAR T-cell therapy for aggressive diseases requires a combination of scientific innovation, process and supply chain op?imization, alternative pathways for care and quality control measures to ensure timely and effective treatment while prioritizing patient safety (**Figure 1**).

## Author contributions

FM-O: Writing – original draft, Writing – review & editing. AG-D: Writing – original draft, Writing – review & editing. NG: Conceptualization, Writing – original draft, Writing – review & editing.
